# Endothelin receptor antagonists in diabetic and non-diabetic chronic kidney disease

**DOI:** 10.1093/ckj/sfae072

**Published:** 2024-03-19

**Authors:** Vanja Ivković, Annette Bruchfeld

**Affiliations:** University Hospital Center Zagreb, Department of Nephrology, Hypertension, Dialysis and Transplantation, Zagreb, Croatia; University of Rijeka, Faculty of Health Studies, Rijeka, Croatia; Department of Health, Medicine and Caring Sciences, Linköping University, Linköping, Sweden; Department of Renal Medicine, Karolinska University Hospital and CLINTEC Karolinska Institutet, Stockholm, Sweden

**Keywords:** chronic kidney disease, drugs, endothelin, endothelin receptor antagonists, review

## Abstract

Chronic kidney disease (CKD) is one of the major causes of morbidity and mortality, affecting >800 million persons globally. While we still lack efficient, targeted therapies addressing the major underlying pathophysiologic processes in CKD, findings of several recent trials have brought about a shifting landscape of promising therapies. The endothelin system has been implicated in the pathophysiology of CKD and endothelin receptor antagonists are one class of drugs for which we have increasing evidence of efficacy in these patients. In this review we summarize the most recent findings on the safety and efficacy of endothelin receptor antagonists in diabetic and non-diabetic CKD, future directions of research and upcoming treatments.

## INTRODUCTION

Chronic kidney disease (CKD) is an umbrella term for several different kidney diseases causing long-term damage and kidney function decline. CKD affects >800 million persons globally and is one of the few non-communicable diseases increasing in prevalence. By 2040, CKD is expected to be the fifth most common cause of death [1, 2]. Diabetic kidney disease, which is strongly linked to the rising prevalence of type 2 diabetes mellitus (T2DM), occurs in up to 50% of patients with CKD [3]. While we currently lack efficient targeted therapies addressing the major underlying pathophysiologic processes in CKD—chronic inflammation and fibrosis—several recent major trials have brought about a shifting landscape of promising therapies. One class of drugs for which there is increasing evidence is endothelin receptor antagonists. Endothelin is a polypeptide produced mainly in the glomerular endothelial cells, which binds to the ET_A_ receptor and causes vasoconstriction and an increase in blood pressure [4, 5]. Endothelin-1 causes vasoconstriction in both renal afferent and efferent arterioles, but it is still not completely elucidated which segment exhibits greater sensitivity, and the issue on the potency of activity on each segment, especially in different complex pathophysiological scenarios, remains controversial [6]. Sustained vasoconstriction of the renal afferent arteriole results in a decrease in the glomerular filtration rate (GFR), podocyte damage and subsequent proteinuria. Furthermore, in some diseases, such as diabetes mellitus, endothelin-1 secretion might promote more pronounced efferent arteriolar constriction, which in concert with other vascular factors and tubular signals affecting the afferent and efferent arterioles, might lead to glomerular hyperfiltration [7]. ET_A_ has therefore been perceived favourably as a potential therapeutic target for selective blockade that may achieve alleviation of hypertension and kidney disease [4].

In this review we plan to summarize the most recent findings on the safety and efficacy of endothelin antagonists in patients with diabetic and non-diabetic CKD, including glomerular disease, and propose ideas for future research.

### Pathophysiology and mechanisms of action

Endothelins are a group of polypeptides forming a major part of the axis involved in vasoconstriction [8]. They have three major isoforms: endothelin-1, endothelin-2 and endothelin-3, with endothelin-1 being the strongest endogenous vasoconstrictor and is most probably of greater clinical importance than the two other isoforms [9]. Binding of endothelin-1 to ET_A_ promotes vasoconstriction, cell proliferation and matrix accumulation, while binding to ET_B_ leads to vasodilation and antiproliferative and antifibrotic effects. These effects are primarily achieved locally, i.e. in an autocrine and paracrine way [10].

Endothelins are also involved in nociception, as endothelin-1 has been shown to have a role in pain transmission, activating nociceptors and potentiating the effect of other algogens [11, 12]. This effect has recently been explored in a post hoc analysis of SONAR, a pivotal trial on atrasentan, which showed that, compared with placebo, patients receiving atrasentan initiated fewer analgesics, including non-steroidal anti-inflammatory drugs (NSAIDs), and had fewer pain-related adverse events (AEs) [13]. The potential for pain reduction, but also the indirect link with less NSAID nephrotoxicity, is a promising additional benefit and needs to be explored in future trials.

**Figure 1:**
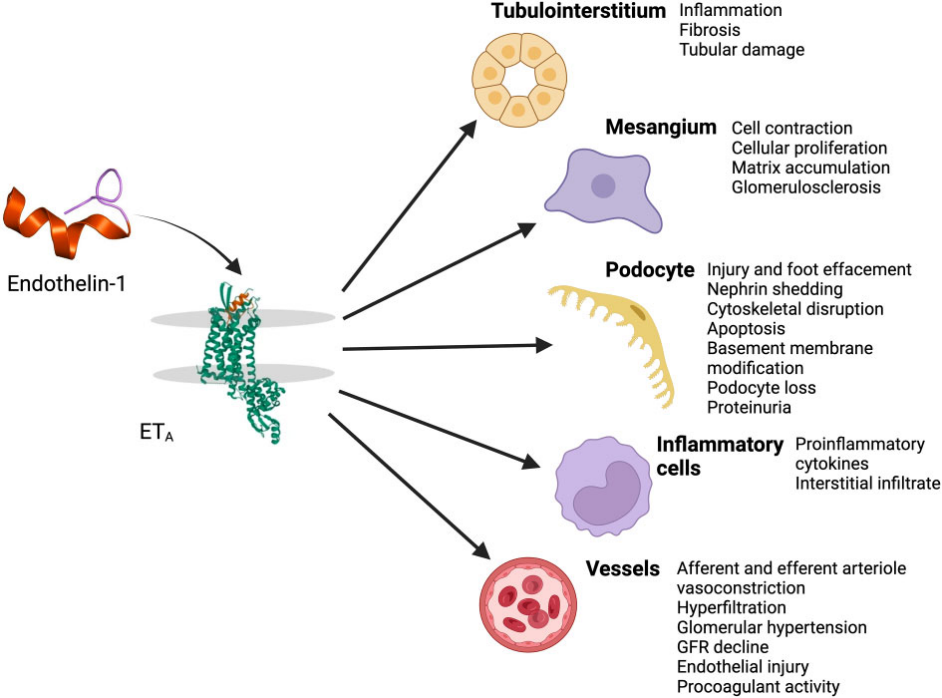
Endothelin-1 actions in the kidney contributing to the development of CKD.

Endothelial dysfunction, of which increased production of endothelins is a key feature, is associated with high cardiovascular and renal risk in CKD patients [14]. Experimental and clinical studies suggested early on that endothelin-1 increases insulin resistance, a pathophysiological hallmark of type 2 diabetes characterized by reduced insulin sensitivity, and hyperglycaemia [15, 16]. Furthermore, urinary biomarkers associated with increased endothelin-1 levels have been shown in all causes of CKD, including diabetes, hypertension, glomerular disease and polycystic kidney disease [17]. Endothelin-1 is causally implicated in several detrimental effects in different parts of the kidney, including enhanced vasoreactivity and procoagulant activity leading to vascular injury, nephron shedding, disruption of the podocyte cytoskeleton and proteinuria leading to podocyte injury, inflammation, tubulointerstitial fibrosis and mesangial cell proliferation and accumulation of extracellular matrix [17, 18] (Fig. 1). An increase in endothelin-1 may also lead to the production of other vasoconstrictors, most notably angiotensin II, which can in turn lead to a positive feedback loop resulting in higher renal endothelin-1 production, further contributing to the damage [19, 20].

The mechanism of action of ET_A_-selective antagonists has been extensively studied and involves several haemodynamic changes, most notably an increase in renal blood flow, but only in patients with CKD and hypertension in whom ET_A_ activity in the kidney is increased, while ET_B_-selective antagonists led to generally opposite effects [5]. Further studies also demonstrated an effect on proteinuria, reducing it in non-diabetic CKD patients and showing that the effects of ET_A_-selective antagonists are highest in the presence of maximal renin–angiotensin system (RAS) blockade [21, 22]. These findings have led to considerable interest and subsequent clinical studies of ET_A_ blockade in CKD.

### Safety and efficacy in diabetic CKD

The first clinical data on the effect of ET_A_ antagonists emerged from a large set of patients in the ASCEND trial of avosentan versus placebo in patients with diabetic nephropathy [23]. The trial showed that avosentan decreased the urinary albumin:creatinine ratio (UACR) but was terminated prematurely after a median follow-up of 4 months due to an excess of cardiovascular events in the avosentan group. AEs were more frequent in the avosentan group, especially fluid overload and congestive heart failure. Atrasentan, which has a much higher affinity for ET_A_, was first tested in two smaller randomized controlled trials (RCTs) that showed clinical efficacy and a relatively good safety profile with ‘manageable fluid retention’ and subsequently in a large RCT, SONAR, whose design included a few caveats to avoid the safety pitfalls encountered in the ASCEND trial [18, 24, 25]. Results of SONAR, along with those of CREDENCE, which evaluated canagliflozin and were published at the same time, were much anticipated, as these were the first successful trials of kidney-targeting therapies in diabetes in more than a decade [26].

This trial enrolled and randomly assigned >2600 patients with type 2 diabetes and an estimated GFR (eGFR) ranging from 25 to 75 ml/min/1.73 m^2^ to either atrasentan or placebo. Random allocation was preceded by a 6-week open-label enrichment period in which all patients received 0.75 mg of atrasentan daily. The aim of the enrichment period was twofold: to select patients without signs of sodium retention (assessed by a weight gain >3 kg or a B-type natriuretic peptide >300 pg/ml) and to separate patients with at least a 30% reduction in UACR (termed responders) from patients with a <30% reduction in UACR (termed non-responders). After this, the responders with no sign of fluid retention were randomized to either atrasentan or placebo. Furthermore, to test if atrasentan could also be of benefit to non-selected patients, the 1020 non-responders who showed no signs of fluid retention and had a UACR reduction >30% were also randomized in a separate stratum to receive either atrasentan or placebo. After a median follow-up of 2.2 years, patients receiving atrasentan had a 35% lower hazard ratio of doubling of serum creatinine or end-stage kidney disease. Safety analysis showed a higher proportion of fluid retention and anaemia in the atrasentan group, but no difference in hospital admission for heart failure or death compared with placebo. A post hoc analysis showed that this effect on eGFR was consistent across baseline UACR and eGFR subgroups and that the absolute risk reduction was highest in the lowest eGFR and highest albuminuria categories, with no difference in the risk of heart failure hospitalization across these subgroups, and that risk for heart failure was associated with baseline BNP and early changes of BNP in response to atrasentan [27, 28].

While the study was not sufficiently powered to detect outcomes in non-responders or compare outcomes between responders and non-responders, atrasentan effects were consistent when responders and non-responders were analysed together, which might be a consequence of the regression to the mean or the threshold of 30% UACR reduction being suboptimal [29]. Despite these promising kidney outcomes, atrasentan did not provide any cardiovascular advantage, as there were no differences in the secondary cardiovascular composite endpoint consisting of cardiovascular death, non-fatal myocardial infarction or non-fatal stroke [30]. Importantly, SONAR was stopped early due to slow recruitment, recruiting only around half of the planned number of patients, and achieving only half of the predicted outcome events [31].

The trial focusing on diabetic kidney disease had a novel research design by introducing some tenets of personalized medicine by the pre-selection of responders, underlying the importance of careful selection of patients both in trials and in clinical practice. While this design did mimic clinical practice to some degree, in which discontinuation and subsequent changes in drugs are frequent in patients who do not show an improvement to initial therapy, it is not certain that this would be reliably reflected in the clinical setting. While there was no difference in treatment or follow-up based on response after randomization, there are some open questions whether this approach could introduce a bias. Given that such responders, defined in this study by both a cut-off of a minimum 30% reduction in proteinuria which is considered a minimum threshold associated with improved renal outcome, and a lack of any signs of fluid retention, have been shown in prior studies to have a lower risk of CKD progression and have already been shown to tolerate the drug.. While proteinuria is strongly associated with eGFR decline, it is not always certain that this relationship is causal or dependent on the same mechanisms [32–34]. Furthermore, even in this population that showed good tolerance of low-dose atrasentan during the brief duration of the enrichment period and despite the use of diuretics, 36.6% of patients who continued using atrasentan in the post-randomization phase had fluid overload and 5.5% had heart failure. This further emphasizes the important message of the SONAR trial pertaining to other endothelin receptor antagonists, that finding the right (lowest effective) dose is crucial and that vigilant monitoring for AEs is warranted, especially in wider clinical use.

While there is wide agreement that the design is both innovative and valid, Walsh [31] postulated that the findings may not be robust and that the study had a low fragility index, i.e. that only one extra event in the atrasentan group would shift significance from *P* < .005 to *P* ≥ .05, but the authors provided a recalculation using individual-patient data that disproved this and showed that at least 11 such additional events are needed to achieve this and that the fragility index of this trial was similar or better than that of a large number of recent trials [31, 35]. Moreover, there were doubts of external validity, as around half of the screened patients entered the enrichment period, which further halved this population to only a quarter of patients screened initially and showing response to therapy, which casts doubt on the broader applicability of atrasentan [36].

Data collected during the SONAR trial have been extensively analysed, resulting in a series of post hoc analyses that has further deepened the insights of the original trial, provided more granular findings in different subpopulations, redefined kidney outcomes and endpoints for future trials and added data on novel associations. A prespecified analysis showed that UACR response to atrasentan achieved during the enrichment phase persisted throughout the remainder of the study and predicted the primary kidney outcome. Furthermore, UACR levels in the placebo group remained below the pre-enrichment phase in the two highest urinary response strata, but not in the two lowest strata. Consequently, early UACR response to enrichment-phase atrasentan was associated with a lower incidence of primary kidney outcome even during placebo. The predictive effect of early albuminuria changes during atrasentan were eliminated after placebo correction, which led to a consistent relative risk reduction with atrasentan compared with placebo independent of initial UACR response. These results were unexpected and it is not clear why the association between albuminuria changes during the enrichment phase and kidney outcomes observed in the atrasentan group were eliminated after placebo correction. One possible explanation is that albuminuria change is not causally associated with the long-term effect of atrasentan and that other factors unrelated to the early UACR response to atrasentan might explain the lower risk of kidney outcomes. Another possibility is that an early change in UACR predicts long-term benefit of atrasentan, but other issues related to imprecise assessment of UACR, regression to the mean and introduction of concomitant medication during enrichment might have prevented the authors from confirming this predictive effect. It is unclear why albuminuria did not return to baseline in the placebo group and the authors postulated that this might be due to several reasons: 1) the potential effect of regression to the mean as UACR levels in the highest response strata of the placebo group remained below pre-enrichment values while those in the lowest response strata exceeded pre-enrichment levels so that the measured albuminuria values at baseline could be higher than the actual values in some responders and those in the non-responders might be lower than the actual values, which could result in an overestimation or underestimation, respectively. 2) Alternatively, atrasentan might have a longer-lasting effect than expected. UACR fluctuates within individuals over time, which might complicate determination of the true change in levels and preclude the separation of responders and non-responders. 3) A higher number of patients in higher UACR response strata may have initiated diuretic therapy during the enrichment period, which might have potentiated the effects of RAS.

This analysis also evaluated the pretrial rate of eGFR decline and found similar rates between albuminuria responders and non-responders, which confirmed that intrinsic differences in patient characteristics are an unlikely explanation of trial outcomes, i.e. that patients receiving atrasentan did not possess any intrinsic, genuine lower risk of CKD progression [37]. Coupled with the population of the RADAR trial, another post hoc analysis has also provided further clinical evidence that endothelin receptor blockade is related to insulin resistance, confirming not only that insulin resistance is associated with adverse cardiorenal outcomes, but also that atrasentan reduced homeostatic model assessment for insulin resistance in a dose-dependent manner by ≈10–25% [38].

A reanalysis of SONAR that aimed at exploring the variability in the effect on eGFR and fluid retention among trial patients undergoing enrichment (run-in phase) found large variations in the median atrasentan area under the concentration–time curve (AUC) as well as in median UACR and BNP. A higher atrasentan AUC was, along with several other patient characteristics such as age, sex, race and body weight, independently associated with a reduction in UACR and an increase in BNP. These partially explained the variability in effect and point to the need to tailor dosing to individual patient characteristics to achieve optimal outcomes [39]. Another potential modifying factor is genetics, as *OATP1B1* gene polymorphisms encoding a hepatic organic anion transporter, have been shown to affect between-patient variability in atrasentan plasma exposure and to be associated with long-term efficacy and safety [40]. A pharmacokinetic reanalysis quantifying the dose–response relationships with efficacy endpoints and safety events showed that atrasentan 0.75 mg/day provided a mean exposure that translated to a reduction of 24% in the hazard of kidney events and an increase of 13% in the hazard of heart failure, confirming the good risk–benefit profile [41].

Given the high proportion of patients with fluid overload and heart failure, the idea of combining endothelin receptor antagonists with sodium–glucose co-transporter-2 inhibitors (SGLT2is) to achieve a diuretic and synergistic nephroprotective effect came early. A post hoc analysis of SONAR showed that combined treatment with SGLTi and atrasentan versus atrasentan alone demonstrated decreased body weight, a surrogate endpoint for fluid retention, and further decreased albuminuria, mitigating risks and decreasing the risk of mortality, which is tightly linked to residual albuminuria [42, 43]. Further experimental evidence confirmed that combining ET_A_ receptor antagonists with SGLT2i attenuates ET_A_ receptor antagonist–induced fluid retention in 4% in salt-fed WKY rats [44]. This was followed by the recently published ZENITH-CKD Phase 2b RCT comparing zibotentan, a potent and highly selective ET_A_ antagonist, with placebo in diabetic and non-diabetic CKD patients [45, 46]. ZENITH-CKD was planned as a two-part study. Part A would enrol patients in four treatment arms: zibotentan 5 mg and dapagliflozin 10 mg, zibotentan 5 mg, dapagliflozin 10 mg or placebo. Part B would include an additional cohort randomized into the aforementioned four treatment arms and two additional arms consisting of zibotentan 0.25 mg and dapagliflozin 10 mg or zibotentan 1.5 mg and dapagliflozin 10 mg. However, an ad hoc safety review closed the randomization in groups receiving high-dose zibotentan (± dapagliflozin) due to the high rate of fluid-retention events, while randomization to the placebo arms was closed due to recent Kidney Disease: Improving Global Outcomes guidelines confirming SGLT2i as the standard of care in the management of CKD [47, 48]. The trial design implemented stratified randomization to ensure that the non-diabetic CKD patients comprise 30–50% of patients in the stratum, approximately reflecting real-life incidences and ensuring a sufficient number of patients with this aetiology of CKD. The trial included 449 patients with a wide range of eGFRs, with around a quarter of patients having eGFRs of 20–30 ml/min/1.73 m^2^ and a third having a UACR >1000 mg/g, and a broad spectrum of CKD aetiology. Roughly half of the patients had diabetic kidney disease, 18.4% had ischaemic/hypertensive nephropathy, 12.3% had glomerulonephritis and the remaining patients had unknown or other aetiologies of CKD.

Patients were randomized and allocated to receive zibotentan 1.5 mg plus dapagliflozin 10 mg, zibotentan 0.25 mg plus dapagliflozin 10 mg or dapagliflozin 10 mg plus placebo, on top of angiotensin-converting enzyme inhibitors (ACEis) or angiotensin receptor blockers (ARBs) if tolerated. At the 12-week follow-up, there was a significant decrease in UACR in both the higher- and lower-dose zibotentan groups (mean difference 33.7% and 27.0%, respectively). Moreover, 18% of patients receiving higher-dose and 9% of patients receiving lower-dose zibotentan plus dapagliflozin had fluid-retention events compared with 8% of those receiving dapagliflozin plus placebo, pointing to a zibotentan dose of 0.25 mg as potentially better in terms of risk–benefit optimization. A subsequent planned trial, ZENITH High Proteinuria (https://clinicaltrials.gov/study/NCT06087835), will be of great interest, as it will include patients with CKD and high levels of proteinuria (eGFR ≥20 and <90 ml/min/1.73 m^2^ and UACR >700 mg/g or urinary protein:creatinine ratio (UPCR) >1000 mg/g).

### Safety and efficacy in non-diabetic CKD

Two major phase 3 RCTs have recently reported on the safety and efficacy of sparsentan in glomerular disease: PROTECT, which enrolled patients with immunoglobulin A nephropathy (IgAN), and DUPLEX, which was the largest RCT to date in focal segmental glomerulosclerosis (FSGS). While both studies confirmed that the safety profile of sparsentan is good and comparable to the active comparator irbesartan, there are several points and notable differences in the efficacy outcomes, especially the effect on eGFR preservation. The recently published final report of the PROTECT study, a randomized, active-controlled, phase 3 trial of sparsentan versus irbesartan in patients with IgAN, showed that sparsentan, a single-molecule, dual endothelin angiotensin receptor antagonist, not only reduces proteinuria by 40% compared with irbesartan, but also maintained this reduction in proteinuria and showed a statistically non-significant trend in slowing down eGFR decline, with a 1.0 ml/min/1.73 m^2^ total slope difference across a 2-year follow-up (*P* = .058). Given the initial eGFR-lowering effect of both sparsentan and irbesartan, the trial also reported the chronic eGFR slope (starting from week 6), which was slightly better (1.1 ml/min/1.73 m^2^) as compared with the total slope and showed marginal statistical significance (*P* = .037) [49].

Analysing the effect of proteinuria, it is important to mention that a higher proportion of patients receiving sparsentan achieved the current target proteinuria level of <1 g/day and, even more importantly given recent evidence on the strong association of low proteinuria and improved outcomes in IgAN and subsequent efforts to lower the proteinuria threshold close to zero, a 2.5-fold greater proportion of sparsentan patients (31%) achieved proteinuria <0.3 g/day [50–52]. Findings of the DUPLEX trial on sparsentan in FSGS are somewhat less convincing given that, despite a similar sustained reduction in proteinuria with a higher proportion of patients achieving partial remission (42.0% versus 26.0%), there was no effect on eGFR decline, with slopes comparable between sparsentan and irbesartan during the 2-year follow-up [53]. This lack of effect on eGFR decline, the primary endpoint of the trial, might be due to several reasons, some of them a consequence of the nature of FSGS itself (e.g. a heterogeneous trial population), i.e. the proportion of cases that might have a genetic as opposed to an immune-mediated disease or the proportion of patients with relapsing disease or advanced CKD and a high percentage of interstitial fibrosis and tubular atrophy. Furthermore, patients in the sparsentan group had a higher initial decrease in eGFR (−4.1 versus −0.8 ml/min/1.73 m^2^) and, as the authors postulate, it was probably difficult for it to ‘catch-up’ to irbesartan through the end of the study. However, when looking at week 6 to the end of the study, there was similarly no difference between the slopes (difference 0.9 ml/min/1.73 m^2^).

The effect on proteinuria was smaller than expected, as the group hypothesized a difference between the proportions of patients achieving partial remission of 30% but achieved only a difference of 16%. This had consequences on detecting differences between eGFR slopes, as the difference was much lower than the 2.5 ml/min/1.73 m^2^ for which the study was powered. Another point that needs be considered is the potential effect of immunosuppressive treatment on eGFR in the irbesartan group as, after excluding all measurements obtained after initiation or intensification of immunosuppressive therapy, the eGFR in the sparsentan group improved. The DUPLEX trial also showed not only a higher proportion (18.5% versus 7.5%), but also earlier occurrence of complete remission with sparsentan, especially important given previous findings of a post hoc analysis of the DUET trial, which reported that achievement of complete remission of FSGS at any time, even if not sustained, is associated with a slower rate of eGFR decline [54]. Antiproteinuric effects reported in DUET have been largely confirmed in DUPLEX and, given the apparent association of complete remission and eGFR decline, there is a need to quantify the relationship of proteinuria and eGFR and develop robust clinical endpoints for use in FSGS.

While the differences in the findings of the efficacy of sparsentan among these two pivotal trials stem from the very different nature of IgAN and FSGS, there are also some other key points common to all glomerular diseases. It is important to note the differential antiproteinuric response between PROTECT (−43% with sparsentan versus −4% with irbesartan) and DUPLEX (−50% with sparsentan versus −32% with irbesartan), which also may have played a role in this. DUPLEX included patients with a somewhat higher eGFR and a much higher baseline proteinuria compared with PROTECT, and a recent study by Collier *et al*. [55] demonstrated that, at least in CKD, disease severity (more severe CKD and higher proteinuria) might affect the chronic slopes. It is important to note that the eGFR slope, a key endpoint in these RCTs and a frequent surrogate endpoint in CKD studies, has been shown to have greater treatment effect variability in glomerular disease trials versus CKD trials, which may be one of the reasons for the differences in findings between PROTECT and DUPLEX and showcases the necessity for a higher number of patients in glomerular disease trials or longer follow-up [56]. One potential resolution to at least a part of this problem, especially in future trials evaluating not only longitudinal eGFR changes, but also major clinical kidney outcomes, might be a new hierarchical endpoint recently proposed by Heerspink *et al*. that led to higher statistical power in a reanalysis of seven major CKD trials at equivalent sample sizes [57, 58]. Their previous research showed that the relative effects of new nephroprotective therapies, i.e. canagliflozin, dapagliflozin, finerenone and atrasentan, are consistent and similar across different endpoints based on varying eGFR decrease thresholds (40%, 50% or 57% eGFR reduction), which might lead to clinical trials that would require significantly fewer patients to achieve equivalent statistical power [59].

Zibotentan has also shown promise in a phase II study of systemic sclerosis–associated CKD consisting of three substudies: ZEBRA 1, an RCT in systemic sclerosis patients with CKD stages 2–3, in which zibotentan 10 mg was given (with a possibility of a reduction to 5 mg in case of fluid retention) with matched placebo; ZEBRA 2A, a placebo-controlled trial in scleroderma renal crisis patients not requiring dialysis, in which zibotentan 2.5 mg was given and increased weekly to a maximum dose of 10 mg with matched placebo; and ZEBRA 2B, an open-label pharmacokinetic study in dialysis patients in which zibotentan 2.5 mg was given and increased to a maximum dose of 10 mg. In patients with CKD stages 2–3, a feasible dose regimen for dialysis patients was found that can be extended in future studies, which are relatively scarce in end-stage kidney disease [60].

## CONCLUSIONS AND FUTURE CONSIDERATIONS

In this review we summarized the main findings on the safety and efficacy of endothelin receptor antagonists in diabetic and non-diabetic CKD. The abundance of evidence stemming from several recent large RCTs confirms them as key therapeutic options for slowing down the progression of CKD. While several safety concerns did put a temporary halt on the widespread use of some earlier endothelin receptor antagonists, ZENITH-CKD proved that a highly selective ET_A_ antagonist combined with an SGLT2i is a safe and efficacious therapy for slowing down eGFR decline and reducing albuminuria in both diabetic and non-diabetic CKD.

Further research on the efficacy of endothelin receptor antagonists in patients with CKD and high proteinuria and with glomerular disease is much warranted and currently under way (Table 1). This is especially important given the results of the sparsentan trials and the generalizability of findings and good effect of SGLT2is in IgAN and FSGS [61, 62]. The combination of endothelin receptor antagonist with SGLT2i and potentially angiotensin II receptor antagonist in a form of ‘triple therapy’ might reduce adverse events and potentially provide a synergistic effect on the efficacy outcomes and is akin to the Four Pillars paradigm, a combination of SGLT2i, GLP-1 receptor agonist and non-steroidal mineralocorticoid receptor antagonist on top of ACEi/ARB in the cardiology field, which has recently been shown to reduce the risk of major adverse cardiovascular endpoints (MACE) by one-third and provide 3.2 years free of MACE and 5.5 years free of CKD progression in patients with type 2 diabetes and albuminuria [63].

**Table 1:** Major studies on endothelin receptor antagonists in diabetic and non-diabetic CKD currently in the pipeline

Study name (acronym) ID	Phase	Study population	Drugs	Planned enrolment	Outcomes (primary; secondary and others)
Atrasentan in Patients With IgA Nephropathy (ALIGN); NCT04573478	Phase 3	Biopsy-proven IgA nephropathy	Atrasentan versus placebo (all receiving RAS inhibitors, some receiving background SGLT2i)	320 subjects	Change in proteinuria (UPCR) Change in GFR Composite endpoints (reduction in eGFR, dialysis, transplantation, mortality) Change in proteinuria (24-h collection)
Randomized, Double-blind, Placebo-controlled, Crossover Study of Atrasentan in Subjects With IgA Nephropathy (ASSIST); NCT05834738	Phase 2	Biopsy-proven IgAN	Atrasentan versus placebo (both on a background SGLT2i and RAS inhibitors)	52 subjects	Change in proteinuria (UPCR) Change in proteinuria at 24 weeks of treatment (UPCR)
Atrasentan in Patients With Proteinuric Glomerular Diseases (AFFINITY); NCT04573920	Phase 2	Proteinuric glomerular diseases (IgAN, FSGS, Alport syndrome, diabetic kidney disease)	Atrasentan (all receiving RAS inhibitor; diabetic kidney disease patients receiving SGLT2i)	100 subjects	Change in proteinuria (IgAN, FSGS and Alport patients) Change in albuminuria (diabetic kidney disease patients)
Zibotentan and Dapagliflozin in Patients With Type 2 Diabetes and Elevated Albuminuria (ZODIAC); NCT05570305	Phase 2	Type 2 diabetes mellitus with albuminuria	Zibotentan and dapagliflozin versus placebo (all receiving RAS inhibitor)	38 subjects	Change from baseline in albuminuria after 4 weeks combined zibotentan and dapagliflozin treatment versus 4 weeks treatment with zibotentan alone Changes in extracellular fluid, body weight, N-terminal prohormone of brain natriuretic peptide, GFR, haematocrit, systolic blood pressure, diastolic blood pressure, renin–angiotensin–aldosterone system markers, copeptin
Study to Investigate Efficacy, Safety, and Tolerability of Zibotentan/Dapagliflozin Compared to Dapagliflozin in Participants With Chronic Kidney Disease and High Proteinuria (ZENITH High Proteinuria); NCT06087835	Phase 3	CKD with high proteinuria (UACR >700 mg/g or UPCR >1000 mg/g)	Zibotentan and dapagliflozin versus dapagliflozin (all receiving RAS inhibitor)	1500 subjects	Change in GFR from baseline Change in UPCR Change in UACR Time to any component of the composite endpoint (eGFR decline, ESRD, death) Change in systolic blood pressure Proportion of participants achieving UPCR <1000 mg/g and >30% reduction
A Study to Investigate Safety and Effect of Sparsentan in Combination With SGLT2 Inhibition in Participants With IgAN (SPARTACUS); NCT05856760	Phase 2	Biopsy-proven IgAN	Sparsentan (all receiving background SGLT2i)	60 subjects	Change in UACR UACR <0.2 g/g Reduction in UACR (30% and 50%) UACR and UPCR at each visit eGFR at each visit Blood pressure at each visit
A Study of the Safety and Activity of Sparsentan for the Treatment of Incident Patients With Immunoglobulin A Nephropathy (SPARTAN); NCT04663204	Phase 2	Incident biopsy-proven IgAN	Sparsentan	12 subjects	UPCR eGFR Change in proteinuria (24-h) Abnormalities in clinical laboratory assessments and vital signs Incidence of AEs, serious AEs, AEs leading to discontinuation, AEs leading to death
ETA and AT1 Antagonism in ANCA-vasculitis (SPARVASC); NCT05630612	Phase 2	ANCA-associated vasculitis in remission	Sparsentan versus irbesartan	40 subjects	Forearm blood flow Fibrinolytic capacity Blood pressure Arterial stiffness Systemic haemodynamics Proteinuria

Moreover, it will be interesting to test the combination of zibotentan, or another endothelin receptor antagonist, with SGLT2is, in glomerular disease, and the results of the ZENITH High Proteinuria trial will be a first step. Results of the ALIGN study (https://clinicaltrials.gov/study/NCT04573478) examining the safety and efficacy of atrasentan in biopsy-proven IgAN are expected and two other studies in similar groups of patients, an open-label study (ASSIST; https://clinicaltrials.gov/study/NCT05834738) and a crossover RCT (AFFINITY; https://clinicaltrials.gov/study/NCT04573920), will also shed some light on this.

The last several years have brought widely available drugs used to halt the progression of CKD and endothelin receptor antagonists have been shown to have an important place in this expanding armamentarium. Until the dawn of specific therapies targeting kidney fibrosis and their widespread use, these drugs form a major component of the approach to reduce the global burden of CKD.

## FUNDING

None declared.

## AUTHORS’ CONTRIBUTIONS

V.I. and A.K. wrote the manuscript.

## DATA AVAILABILITY STATEMENT

No new data were generated or analysed in support of this research.

## CONFLICT OF INTEREST STATEMENT

None declared.
